# Prognostic value of Kinesin‐4 family genes mRNA expression in early‐stage pancreatic ductal adenocarcinoma patients after pancreaticoduodenectomy

**DOI:** 10.1002/cam4.2524

**Published:** 2019-09-06

**Authors:** Quanfa Han, Chuangye Han, Xiwen Liao, Ketuan Huang, Xiangkun Wang, Tingdong Yu, Chengkun Yang, Guanghui Li, Bowen Han, Guangzhi Zhu, Zhengqian Liu, Xin Zhou, Junqi Liu, Hao Su, Liming Shang, Tao Peng, Xinping Ye

**Affiliations:** ^1^ Department of Hepatobiliary Surgery The First Affiliated Hospital of Guangxi Medical University Nanning People's Republic of China

**Keywords:** biomarker, Kinesin‐4 family genes, pancreatic ductal adenocarcinoma, prognosis

## Abstract

**Background:**

The aim of this study was to investigate the potential prognostic value of Kinesin‐4 family genes mRNA expression in early‐stage pancreatic ductal adenocarcinoma (PDAC) patients after pancreaticoduodenectomy.

**Methods:**

Kaplan‐Meier survival analysis method with log‐rank test and Cox proportional hazards regression analysis were performed to figure out the association between Kinesin‐4 family genes expression and PDAC patients overall survival time. Joint‐effect survival analysis and stratified survival analysis were carried out to assess the prognosis prediction value of prognosis‐related gene. Nomogram was constructed for the individualized prognosis prediction. In addition, we had used the gene set enrichment analysis and genome‐wide co‐expression analysis to further explore the potential mechanism.

**Results:**

*KIF21A* expression level was significantly associated with PDAC patient clinical prognosis outcome and patient with a high expression of *KIF21A* would have a shorter overall survival time. The prognosis prediction significance of *KIF21A* was well validated by the joint‐effect survival analysis, stratified survival analysis, and nomogram. Meanwhile, the gene set enrichment analysis and genome‐wide co‐expression analysis revealed that *KIF21A* might involve in DNA damage and repair, transcription and translation process, post‐translation protein modification, cell cycle, carcinogensis genes and pathways.

**Conclusions:**

Our current research demonstrated that *KIF21A* could serve as a potential prognostic biomarker for patient with early‐stage PDAC after pancreaticoduodenectomy.

## Graphical abstract

In this study, we investigated the potential prognostic value of Kinesin‐4 family genes mRNA expression in early‐stage pancreatic ductal adenocarcinoma patients after pancreaticoduodenectomy. We established the prognostic significance of KIF21A and inferred the possible mechanism by gene set enrichment analysis and genome‐wide co‐expression analysis.

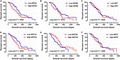

## 1 INTRODUCTION

Pancreatic cancer (PC) is the seventh leading cause of cancer‐related death worldwide, with about 458 918 new cases and 432 242 deaths in 2018 alone.^1^ Furthermore, it was estimated that about 90 100 newly diagnosed PC cases and 79 400 death cases were recorded in China in 2015.^2^ The incidence rate and mortality rate of PC were 10.9/10^5^ and 8.4/10^5^, respectively.^3^ Previous research has reported that PC with a very poor prognosis and the age‐standardized 5‐year relative survival rate for PC was 11.7% in China.^4^ The low survival rate is partly attributed to most patients had no symptom until the disease develops to an advanced stage that will ultimately lead to the patients at a late stage when diagnosed.^5^ Pancreatic ductal adenocarcinoma (PDAC) is the most common histological type of PC, accounting for more than 80% of all pancreatic neoplasms.^6^ Several risk factors are considered to be significantly associated with the development and progression of PC, including cigarette smoking,^7^, ^8^ alcohol consumption,^9^ chronic pancreatitis,^10^, ^11^ diabetes mellitus,^12^, ^13^ obesity,^14^ and a family history of pancreatic cancer.^15^ Surgical resection is at present the only potentially curative therapy strategy that can significantly prolong patient survival time.^5^ Currently, the surgical resection techniques for PC include pancreaticoduodenectomy, distal pancreatectomy with splenectomy, and total pancreatectomy.^5^ The pancreaticoduodenectomy is needed to remove tumors in the head and neck of pancreas and distal pancreatectomy with splenectomy that resects tumors in the body or tail of pancreas.^16^

Kinesin superfamily (KIF) genes consist of more than 40 members that are classified into 14 families (Kinesin‐1 to Kinesin‐14 family).^17^, ^18^ Kinesin‐4 family genes comprise 6 members (*KIF4A*, *KIF4B*, *KIF7*, *KIF21A*, *KIF21B*, and *KIF27*).^19^ Numerous studies had proved that the Kinesin‐4 family genes were notably related to several diseases. *KIF4A* expression was significantly associated with the prognosis outcome of prostate cancer,^20^ breast cancer,^21^, ^22^ lung cancer,^23^ colorectal carcinoma,^24^ and hepatocellular carcinoma.^25^ Li et al found that *KIF7* regulated Gli2 localization and activity in the Hedgehog signaling pathway during the formation of basal cell carcinoma.^26^ In addition, researchers had demonstrated that the missense mutation in *KIF21A* could cause congenital fibrosis of the extraocular muscles.^27^, ^28^, ^29^ Meanwhile, its expression level affected the axonal transport and nervous system development in patients with Down syndrome.^30^ Finally, it was important to note that the expression of *KIF21B* could predict the prognosis of patients with renal cell carcinoma 31 and multiple myeloma.^32^

Given the poor prognosis of PC, especially for the late stage patient, it is very imperative to find more sensitive biomarkers to predict clinical prognosis outcome in the early time. So that we could take effective intervention measures in the early‐stage to improve this gloomy situation. By retrieving the relevant literature, we found that Kinesin‐4 family genes played a crucial role in cancer prognosis and treatment. More importantly, Kinesin‐4 family genes could affect tumor biological behavior, such as proliferation, invasion, metastasis, and so on.^22^, ^23^, ^24^, ^25^ It is well known that the poor prognosis of tumor depends largely on its malignant behavior. So, we speculated that Kinesin‐4 family genes might be associated with pancreatic cancer prognosis. The aim of this study was to explore the potential prognostic value of Kinesin‐4 family genes mRNA expression in early‐stage PDAC patients after pancreaticoduodenectomy established on the public resource and bioinformatic analysis.

## 2 MATERIAL AND METHODS

### 2.1 Bioinformatic analysis of Kinesin‐4 family genes

Gene enrichment analysis including Gene Ontology (GO) function analysis and Kyoto Encyclopedia of Genes and Genomes (KEGG) pathway analysis were carried out by the bioinformatics resources Database for Annotation, Visualization and Integrated Discovery (DAVID) v6.8 (https://david.ncifcrf.gov/, accessed March 6, 2019)^33^, ^34^ to investigate the possible biological functions and potential pathways of Kinesin‐4 family genes. Biological Network Gene Ontology (BiNGO) in Cytoscape (version 3.7.1)^35^ was used to further validate the result of GO terms in DAVID. Interaction networks of Kinesin‐4 family genes in gene‐gene and protein‐protein were performed by the Gene Multiple Association Network Integration Algorithm (GeneMANIA) (http://genemania.org/, accessed March 12, 2019)^36^, ^37^ and the Search Tool for the Retrieval of Interacting Genes/Proteins (STRING) (https://string-db.org/cgi/input.pl, accessed March 12, 2019),^38^, ^39^ respectively. The web‐based tool Gene Expression Profiling Interactive Analysis (GEPIA) (http://gepia.cancer-pku.cn/, accessed March 12, 2019)^40^ was used to compare the expression level of each gene between pancreatic adenocarcinoma (PAAD) tumor tissue and normal tissue by the unpaired *t* test.

### 2.2 Patient information in TCGA database

The clinicopathologic information and corresponding gene expression level of patients were obtained from public database The Cancer Genome Atlas (TCGA) (https://portal.gdc.cancer.gov/, accessed March 2, 2019), and the raw data were normalized by DESeq^41^, ^42^ In order to enhance the reliability of our research conclusion, patient inclusion criteria and exclusion criteria were established as in our previous article.^43^ Briefly, the inclusion criteria were as follows: (a) patient with complete survival information; (b) the histological type was PDAC; (c) the pathologic stage was I or II; (d) patient underwent pancreaticoduodenectomy. In our research, PDAC patients with III or IV stage or underwent other surgical resection techniques were excluded. Based on these criteria, a total of 112 patients were enrolled into this study for the prognosis analysis. The clinical data of the patients including age, gender, alcohol history, histologic grade, pathologic stage, radical resection, radiation therapy, targeted molecular therapy, survival time, and survival statue. The dataset included in this study was downloaded from TCGA public database, approval from the ethics committee was not required.

### 2.3 Survival analysis

Kaplan‐Meier survival analysis method with log‐rank test was used to evaluate the association between clinicopathologic parameters and patient overall survival (OS) time. Log‐rank *P* < .05 was considered to indicate statistical significance and the clinicopathologic feature was identified as the prognosis‐related factor. In order to explore whether the expression level of *KIF4A*, *KIF4B*, *KIF7*, *KIF21A*, *KIF21B*, and *KIF27* were notably connected with patient prognosis outcome, the patients were divided into two groups (low expression group and high expression group) according to the median value of gene expression level in tumor tissue. The cut‐off value is the median value of each gene expression level according to the gene sequencing result. Multivariate Cox regression analysis was performed after adjusting for the prognosis‐related factors. Hazard ratio (HR) and 95% confidence interval (CI) were calculated to evaluate the survival difference. Joint‐effect survival analysis for the combination of gene expression level and prognosis‐related factors was used to assess the combined predictive effect on patient prognosis. Stratified survival analysis for the clinicopathological parameters was carried out to further explore the effect of gene expression level on prognosis in each variable.

### 2.4 Prognostic nomogram construction

A nomogram was constructed using all the enrolled patients as the source population, and clinicopathologic factors and prognosis‐related gene to obtain an individualized prognosis prediction. We could predict the survival probability in the future several years for each patient according to their total point by the nomogram.

### 2.5 Gene set enrichment analysis

In order to further investigate the potential mechanism of different expression level of the prognosis‐related gene affected patient clinical survival outcome, the gene set enrichment analysis (GSEA) (http://software.broadinstitute.org/gsea/index.jsp, accessed March 12, 2019)^44^, ^45^ was conducted in our present study. In GSEA, the Molecular Signatures Database (MSigDB) included C2 (c2.all.v6.2.symbols.gmt), C5 (c5.all.v6.2.symbols.gmt), and C6 (c6.all.v6.2.symbols.gmt)^46^, ^47^ were used to explore the potential mechanism. The latest version of MSigDB gene sets were divided into 8 major collections, in which the C2 for curated gene sets, C5 for GO gene sets, and C6 for oncogenic gene sets. The nominal *P* < .05 and false discovery rate < 0.25 were defined as the significantly enriched gene sets.

### 2.6 Genome‐wide co‐expression analysis of prognostic Kinesin‐4 family genes

To further explore the possible function of prognosis‐related gene in PDAC, the genome‐wide co‐expression analysis was carried out in the current research. Pearson correlation coefficient was calculated. The genes with Pearson correlation coefficient > 0.35 and *P* < .05 were considered as the co‐expression genes. The prognosis‐related gene and its co‐expression genes were used to construct the co‐expression network using Cytoscape software (version 3.7.1).^35^ In addition, GO function analysis and KEGG pathway analysis by the DAVID^33^, ^34^ and GO term validation by the BiNGO plugin in the Cytoscape were performed for function assessment.

### 2.7 Statistical analysis

The SPSS software (version 18.0) was used for all the statistical analysis. Survival analysis was performed using the Kaplan‐Meier method with log‐rank test. Cox proportional hazards regression model was carried out for the univariate and multivariate analysis. The prognosis‐related factors were entered into the multivariate Cox regression analysis for adjustment. Hazard ratio and 95% confidence interval were calculated to evaluate the survival difference. The comparison of gene expression level between tumor tissue and normal tissue was performed by using the unpaired *t* test. *P* < .05 was considered to indicate statistical significantly.

## 3 RESULTS

### 3.1 Bioinformatic analysis of Kinesin‐4 family genes

**Figure 1:**
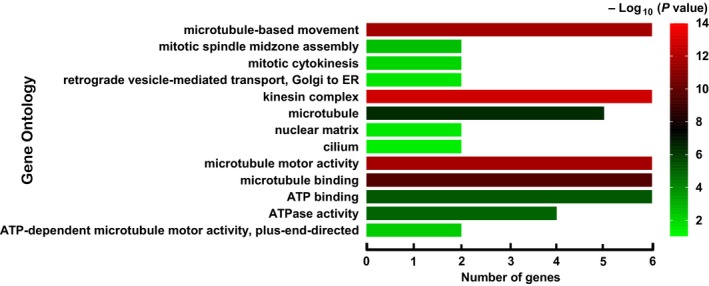
Gene Ontology function enrichment analysis of Kinesin‐4 family genes carried out by the Database for Annotation, Visualization and Integrated Discovery

**Figure 2:**
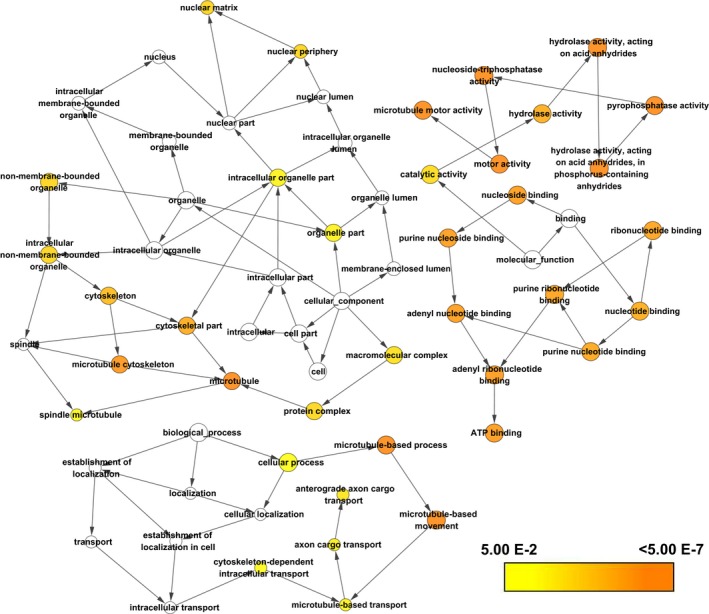
Gene Ontology terms of Kinesin‐4 family genes conducted by Biological Network Gene Ontology in Cytoscape software

**Figure 3:**
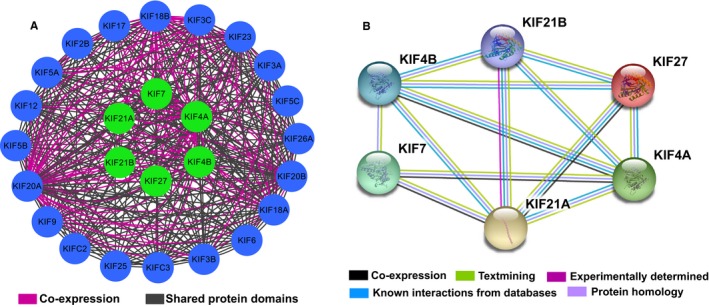
Interaction networks of Kinesin‐4 family genes performed by Gene Multiple Association Network Integration Algorithm (GeneMANIA) and Search Tool for the Retrieval of Interacting Genes/Proteins (STRING). A, Gene–gene interaction network by GeneMANIA. B, Protein–protein interaction network by STRING

**Figure 4:**
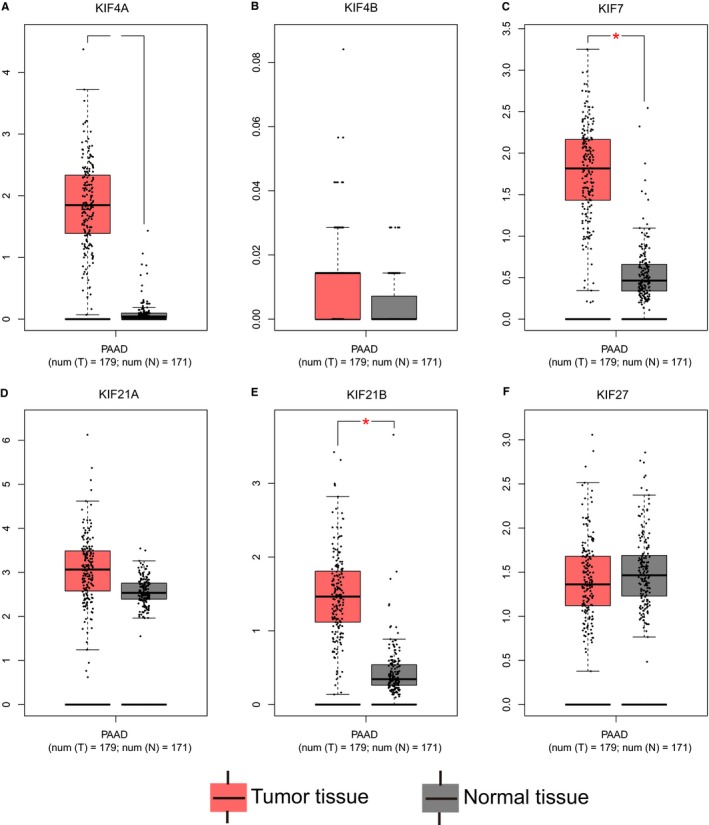
Gene expression level distribution of Kinesin‐4 family genes in pancreatic adenocarcinoma tumor tissue and normal tissue. (A) *KIF4A*; (B) *KIF4B*; (C) *KIF7*; (D) *KIF21A*; (E) *KIF21B*; (F) *KIF27*. *P < .05

The GO function enrichment analysis showed that Kinesin‐4 family genes were mainly enriched in microtubule‐based movement, mitosis process, intracellular transport, microtubule motor activity, and ATPase activity (Figure 1). No potential pathway was observed in the KEGG pathway enrichment analysis. As shown in Figure 2, the result of BiNGO was consistent with the GO term in DAVID. Interaction networks in gene–gene and protein–protein performed by the GeneMANIA and STRING, respectively, showed that *KIF4A*, *KIF4B*, *KIF7*, *KIF21A*, *KIF21B*, and *KIF27* were co‐expressed with each other, interacted within a network, and homologous at the protein level (Figure 3). GEPIA analysis comparing the gene expression level between PAAD tumor tissue and normal tissue showed that *KIF4A*, *KIF7*, and *KIF21B* were significantly upregulated in tumor tissue (*P* < .05), while the expression level for *KIF4B*, *KIF21A*, and *KIF27* were not significantly different between tumor tissue and normal tissue (Figure 4).

### 3.2 Survival analysis

**Figure 5:**
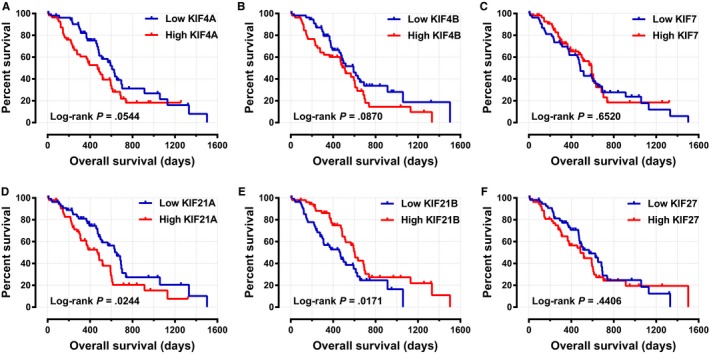
Kaplan‐Meier survival curves of Kinesin‐4 family genes in pancreatic ductal adenocarcinoma patient overall survival. Overall survival stratified by (A) *KIF4A*; (B) *KIF4B*; (C) *KIF7*; (D) *KIF21A*; (E) *KIF21B*; (F) *KIF27*

**Figure 6:**
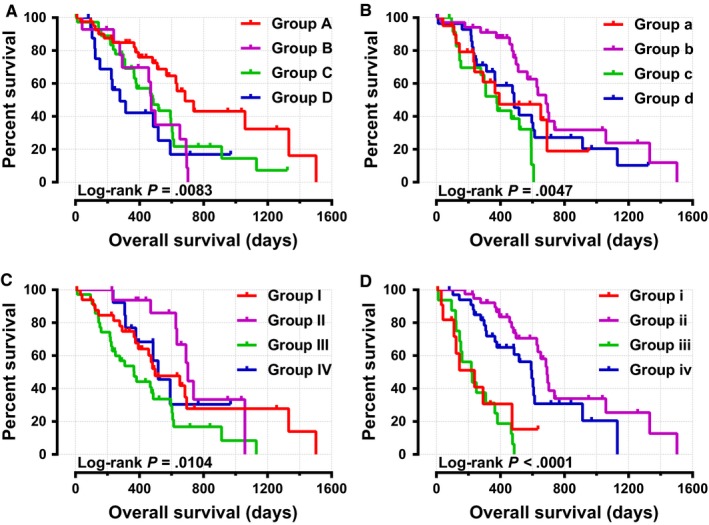
Joint‐effect survival analysis for the combination of *KIF21A* and prognosis‐related clinical factors in pancreatic ductal adenocarcinoma patient overall survival. (A) Histologic grade; (B) Radical resection; (C) Radiation therapy; (D) Targeted molecular therapy

**Figure 7:**
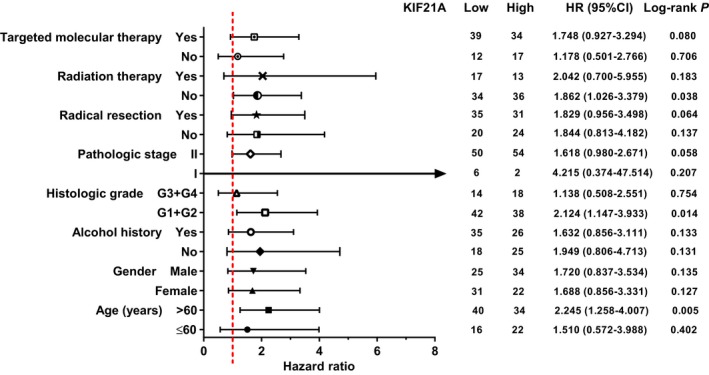
Stratified survival analysis of *KIF21A* in each clinicopathological parameters. HR, hazard ratio; CI, confidence interval

In order to evaluate the association between clinicopathologic parameters and patient OS time, we conducted the Kaplan‐Meier survival analysis with log‐rank test. As shown in Table S1, the tumor histologic grade and patient whether underwent radical resection, radiation therapy, targeted molecular therapy were significantly associated with OS time (all log‐rank *P* < .05). The median survival time of these prognosis‐related factors were histologic grade (596 days vs 470 days), radical resection (381 days vs 603 days), radiation therapy (473 days vs 691 days), and targeted molecular therapy (224 days vs 634 days), respectively. The Cox regression analysis adjusted for the prognosis‐related factors indicated that *KIF21A* expression level was closely connected with patient OS time (adjusted *P* = .020, adjusted HR = 1.876, 95%CI = 1.102‐3.194), and the median survival time for the low and high expression groups were 652 and 476 days, respectively (Table 1 and Figure 5). The joint‐effect survival analysis for the combination of *KIF21A* expression level and each prognosis‐related factor showed better predictive performance for prognosis (Table 2 and Figure 6 **)**. Stratified survival analysis was then carried out to further explore the effect of *KIF21A* expression level on prognosis for each clinicopathological parameters. As shown in Figure 7, high *KIF21A* expression could notably lead to poor clinical prognosis outcome in three subgroups, such as patient age >60 years (*P* = .005, HR = 2.245, 95%CI = 1.258‐4.007), tumor histologic grade was G1/G2 (*P* = .014, HR = 2.124, 95%CI = 1.147‐3.933), and patient who did not underwent radiation therapy (*P* = .038, HR = 1.862, 95%CI = 1.026‐3.379).

**Table 1:** Prognostic value of Kinesin‐4 family genes expression in PDAC patients OS

Gene expression	Patients	No. of events	MST (days)	Crude HR (95%CI)	Crude *P* value	Adjusted HR (95%CI)^a^	Adjusted *P* value^a^
*KIF4A*					.057		.575
Low	56	31	607	1		1	
High	56	38	473	1.606 (0.987‐2.615)		1.163 (0.686‐1.972)	
*KIF4B*					.089		.046
Low	56	32	592	1		1	
High	56	37	511	1.514 (0.938‐2.443)		1.726 (1.011‐2.949)	
*KIF7*					.652		.935
Low	56	40	485	1		1	
High	56	29	592	0.894 (0.549‐1.455)		0.978 (0.573‐1.671)	
*KIF21A*					.026		.020
Low	56	31	652	1		1	
High	56	38	476	1.735 (1.067‐2.820)		1.876 (1.102‐3.194)	
*KIF21B*					.019		.064
Low	56	37	467	1		1	
High	56	32	603	0.555 (0.339‐0.907)		0.595 (0.343‐1.031)	
*KIF27*					.442		.114
Low	56	32	568	1		1	
High	56	37	486	1.206 (0.748‐1.945)		1.526 (0.903‐2.578)	

Abbreviations: CI, confidence interval; KIF, kinesin family; HR, hazard ratio; MST, median survival time; OS, overall survival; PDAC, pancreatic ductal adenocarcinoma.

a Adjusted for histologic grade, radical resection, radiation therapy, targeted molecular therapy.

**Table 2:** Joint‐effect survival analysis of *KIF21A* expression and clinical factors in PDAC patients OS

Group	*KIF21A* expression	Clinical factors	Patients (n = 112)	No. of events	MST (days)	Crude HR (95%CI)	Crude *P* value	Adjusted HR (95%CI)^a^	Adjusted *P* value^a^
Histologic grade									
A	Low	G1 + G2	42	19	684	1	.012	1	.006
B	Low	G3 + G4	14	12	473	2.569 (1.217‐5.422)	.013	2.370 (1.046‐5.367)	.039
C	High	G1 + G2	38	26	486	2.087 (1.130‐3.855)	.019	1.938 (0.956‐3.930)	.067
D	High	G3 + G4	18	12	278	3.109 (1.474‐6.559)	.003	4.242 (1.890‐9.519)	*P* < .001
Radical resection^b^									
a	Low	No	20	11	393	1	.007	1	.023
b	Low	Yes	35	19	684	0.535 (0.248‐1.151)	.110	0.508 (0.223‐1.157)	.107
c	High	No	24	18	378	1.832 (0.851‐3.945)	.122	1.771 (0.785‐3.994)	.168
d	High	Yes	31	20	485	0.965 (0.458‐2.031)	.925	0.993 (0.466‐2.117)	.985
Radiation therapy^c^									
I	Low	No	34	20	498	1	.015	1	.115
II	Low	Yes	17	8	702	0.617 (0.267‐1.426)	.258	0.791 (0.312‐2.008)	.622
III	High	No	36	28	366	1.942 (1.069‐3.526)	.029	1.883 (1.016‐3.489)	.044
IV	High	Yes	13	7	517	0.987 (0.410‐2.380)	.978	1.469 (0.568‐3.796)	.427
Targeted molecular therapy^d^									
i	Low	No	12	8	239	1	*P* < .001	1	*P* < .001
ii	Low	Yes	39	21	691	0.162 (0.068‐0.382)	*P* < .001	0.173 (0.066‐0.454)	*P* < .001
iii	High	No	17	16	219	1.448 (0.612‐3.427)	.400	1.906 (0.787‐4.616)	.153
iv	High	Yes	34	20	596	0.277 (0.119‐0.642)	.003	0.322 (0.130‐0.802)	.015

Abbreviations: KIF21A, kinesin family member 21A; PDAC, pancreatic ductal adenocarcinoma; OS, overall survival; MST, median survival time; HR, hazard ratio; CI, confidence interval.

a Adjusted for histologic grade, radical resection, radiation therapy, targeted molecular therapy.

b Information of radical resection was unavailable in 2 patients.

c Information of radiation therapy was unavailable in 12 patients.

d Information of targeted molecular therapy was unavailable in 10 patients.

### 3.3 Prognosis nomogram construction

**Figure 8:**
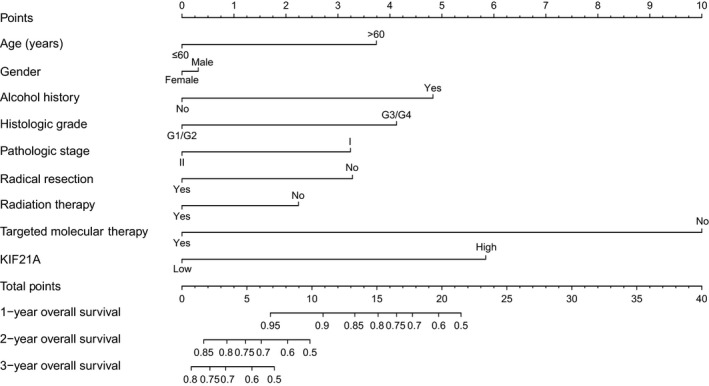
Prognostic nomogram for pancreatic ductal adenocarcinoma patient 1‐, 2‐, 3‐y overall survival prediction

All the clinicopathologic parameters and *KIF21A* expression level were used to develop the nomogram. With each variable was assigned a score, the total point was calculated by summing up these scores of all the variables and located to the scale. We could obtain an individualized prognosis prediction and predict the survival probability in the future several years according to their total points. We performed nomogram analysis for the probabilities of 1‐, 2‐, and 3‐year OS (Figure 8). As shown in the nomogram, the expression level of *KIF21A* contributed to the patient prognosis in some degree.

### 3.4 Gene set enrichment analysis of *KIF21A* in PDAC

**Figure 9:**
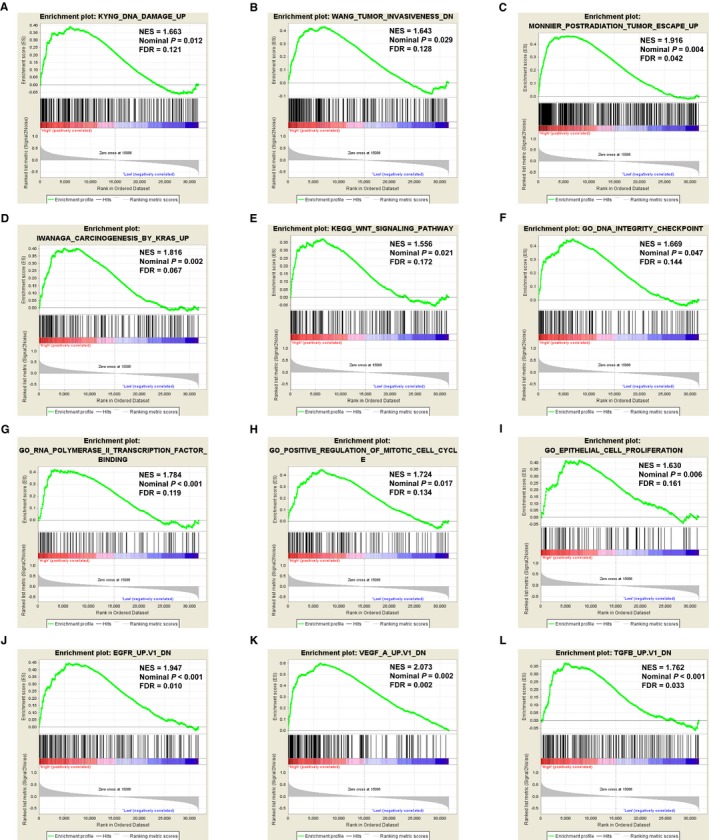
Gene set enrichment analysis (GSEA) of *KIF21A*. (A‐E) GSEA results of C2 gene sets for high *KIF21A* expression groups; (F‐I) GSEA results of C5 gene sets for high *KIF21A* expression groups; (J‐L) GSEA results of C6 gene sets for high *KIF21A* expression groups. NES, normalized enrichment score; FDR, false discovery rate

GSEA was performed to figure out the potential mechanism of different *KIF21A* expression level affected PDAC patient clinical prognosis. In current research, we analyzed the curated gene sets (C2), GO gene sets (C5), and oncogenic gene sets (C6) of the MSigDB. Enrichment of C2 indicated that high expression of *KIF21A* involved in DNA damage, tumor invasiveness, carcinogenesis role of KRAS gene, and WNT pathway (Figure 9A‐E). Enrichment of C5 showed that high expression of *KIF21A* connected with DNA integrity checkpoint, transcription process and cell cycle (Figure 9F‐I). Enrichment of C6 suggested that high expression of *KIF21A* related to various oncogene signatures such as EGFR, VEGF, and TGFB (Figure 9J‐L).

### 3.5 Genome‐wide co‐expression analysis of *KIF21A* in PDAC

**Figure 10:**
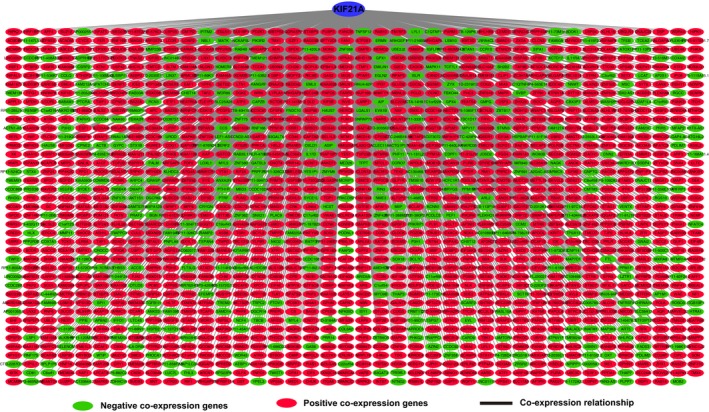
Co‐expression network of *KIF21A* co‐expression genes in pancreatic ductal adenocarcinoma tumor tissue. The green nodes represent negative correlation with *KIF21A*, and the red nodes represent positive correlation with *KIF21A*

**Figure 11:**
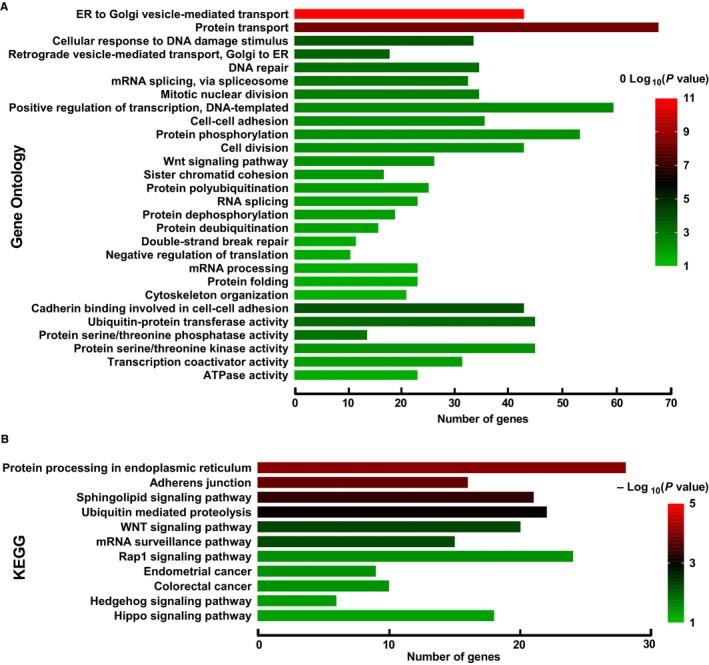
Gene Ontology (GO) function analysis and Kyoto Encyclopedia of Genes and Genomes (KEEG) pathway analysis of *KIF21A* co‐expression genes in pancreatic ductal adenocarcinoma tumor tissue. A, GO function analysis. B, KEGG pathway analysis

Genome‐wide co‐expression analysis was carried out for further exploring possible function of *KIF21A* in PDAC. A total of 1640 genes met the Pearson correlation coefficient > 0.35 and *P* < .05 that were considered as the co‐expression genes. The co‐expression network was constructed which included 449 negative co‐expression genes and 1191 positive co‐expression genes (Figure 10, Table S2). GO function enrichment analysis by DAVID showed that *KIF21A* and its co‐expression genes were mainly enriched in the following biological processes and molecular functions, such as intracellular transport, DNA damage and repair, RNA splicing, cell division, transcription and translation process, protein phosphorylation and ubiquitination (Figure 11A, Table S3). GO term validation result by the BiNGO was consistent with the result of DAVID (Figures [Link], [Link], [Link]). Meanwhile, KEGG pathway enrichment analysis suggested that *KIF21A* and its co‐expression genes were notably enriched in Sphingolipid pathway, WNT pathway, mRNA surveillance pathway, Hedgehog pathway, Hippo pathway and so on (Figure 11B, Table S4).

## 4 DISCUSSION

In our current research, we investigated the relationship between Kinesin‐4 family genes mRNA expression and early‐stage PDAC patient clinical prognosis outcome by collecting data from public resource and performing a series of bioinformatic analysis. We have demonstrated that *KIF21A* expression level was significantly associated with early‐stage PDAC patient overall survival time and patient with a high expression of *KIF21A* would have a shorter overall survival time. So we could conclude that *KIF21A* might serve as a potential prognostic biomarker for early‐stage PDAC patient after pancreaticoduodenectomy. Meanwhile, we further explored the potential mechanism for *KIF21A* mRNA expression level affected PDAC patient prognosis outcome based on the GSEA and genome‐wide co‐expression analysis. As is shown above the potential mechanism might involve in DNA damage and repair, transcription and translation process, post‐translation protein modification, cell cycle, carcinogensis genes, and pathways. However, the exact mechanism still needs more research to further validate in the future.

Kinesin superfamily proteins, as the important molecular motor proteins, played a crucial role in the process of intracellular transport.^48^, ^49^ Furthermore, they had an important function in spindle self‐orgnization and chromosome segregation during mitosis process.^50^, ^51^, ^52^ Kinesin‐4 family genes were involved in a wide range of biological functions and its dysregulation might lead to some pathological processes. Previous studies have reported that *KIF4A* and *KIF4B* participated in the chromosome condensation and segregation, anaphase spindle midzone formation, and cytokinesis.^53^, ^54^, ^55^, ^56^ *KIF4A* was associated with DNA damage response by modulating the BRCA2/Rad51 pathway.^57^ Researchers have demonstrated that during the process of mitosis, loss of *KIF4A* would lead to aneuploidy which ultimately triggered the tumorigenesis.^58^ *KIF4B* and *KIF4A* are two closely related but distinct proteins with over 90% homologous with each other and they play the multiple, possible identical role during mitosis. Recent studies have revealed that *KIF21A* was related to some neuronal diseases. Missense mutation in *KIF21A* could cause congenital fibrosis of the extraocular muscles^27^ and the expression level of *KIF21A* might affect axonal transport and nervous system development in patients with Down syndrome.^30^

Moreover, researchers have reported that Kinesin‐4 family genes played a crucial role in the development, progression, treatment, and prognosis of numerous cancers. *KIF4A* could enhance cell proliferation, promote tumor metastasis, predict patient prognosis and act as the potential therapeutic target in cancer treatment for breast cancer, lung cancer, colorectal cancer, and hepatocellular carcinoma.^22^, ^23^, ^24^, ^25^ Researchers had reported that *KIF21B* as a downstream target gene regulated by the miR‐144‐5p/syndecan‐3 axis which participated in the pathogenesis of renal cell carcinoma and its expression could predict the patient prognosis outcome.^31^ Agnelli et al. reconstructed the gene regulatory networks in multiple myeloma and revealed that *KIF21B* with a prognostic importance could predict the survival of patients.^32^ Numerous researches had proved that the Hedgehog signaling pathway played a crucial role in the development, progression, and therapy of various cancers, such as oesophageal squamous cell carcinoma, gastric cancer, colon cancer, and pancreatic cancer.^59^, ^60^, ^61^, ^62^, ^63^, ^64^ *KIF7* and *KIF27* were both involved in the Hedgehog signaling pathway.^65^, ^66^ Li et al. found that *KIF7* regulated Gli2 localization and activity in the Hedgehog signaling pathway during basal cell carcinogenesis.^26^ In our current research, we demonstrated that high *KIF21A* expression level was significantly associated with the poor prognosis in early‐stage PDAC patients, making it serve as a potential prognostic biomarker for patients with early‐stage PDAC after pancreaticoduodenectomy.

The GSEA and genome‐wide co‐expression analysis were conducted to figure out the potential mechanism of *KIF21A* expression level affect PDAC patient prognosis. As the results showed that, the mechanism might be implicated in several biological processes and signaling pathways, such as DNA damage and repair, transcription and translation process, post‐translation protein modification, cell cycle, carcinogensis genes and pathways (*KARS*, *EGFR*, *VEGF*, WNT pathway, Hedgehog pathway). It is important to note that the above biological processes and pathways are significantly associated with cancer prognosis.^67^, ^68^, ^69^, ^70^, ^71^, ^72^, ^73^, ^74^, ^75^ A number of studies have demonstrated the involvement of several of these processes in pancreatic cancer prognosis. For example, the low expression of *CHD5* could activate DNA damage response and function as useful biomarker for pancreatic cancer poor clinical outcome.^76^ Similarly, phosphorylation status of *IRAK4* was a predictor for postoperative relapse and poor overall survival in patient with PDAC.^77^ Upregulation of *CIAPIN1* could delay cell cycle progression and induce cell apoptosis. The expression level of *CIAPIN1* could act as an independent prognosis factor in pancreatic cancer.^78^ Nectin‐4 gene expression was notably related to *VEGF* expression and intratumoral microvessel density in pancreatic cancer, and therefore its expression level had a significant postoperative prognosis value.^79^ Finally, dysregulation of WNT signaling pathway was significantly connected with lymphvascular invasion and worse survival outcome of pancreatic cancer patients.^80^

To summarize, we have established the prognostic significance of *KIF21A* in early‐stage PDAC patient and inferred the possible mechanism by GSEA and genome‐wide co‐expression analysis. We are committed to obtain a candidate prognosis‐related biomarker for pancreatic cancer and to predict clinical prognosis outcome for the patient. So that we could take effective treatment measures in the early time to improve patient gloomy prognosis. In addition, we also hope to provide an applicable effect target for PDAC therapy. Meanwhile, there are still some limitations in our study that need to be clarified. First, our subject mainly focused on a select group of early‐stage PDAC patient who underwent pancreaticoduodenectomy, so the sample size is limited. Therefore, a large sample size and multi‐center clinical cohort research is needed to enhance the reliability of our conclusion. Second, the GSEA and genome‐wide co‐expression analysis as an analysis approach which just provided inference of the potential mechanism underlying the *KIF21A* expression level affect prognosis outcome. The exact mechanism can only be elucidated with more molecular and functional studies validation in the future. Third, since our research performed by bioinformatics analysis that involved whole genome data, some results that reached statistical significance may be contingent. In addition, the results of our study are only based on a TCGA cohort analysis, lacking of the validation cohort. Therefore, our research results have yet to be further verified in future research. Fourth, our current study was mainly established on collecting data from public resource and performing a series of bioinformatic analysis. So, further experiment validation about the expression, function, and molecular mechanism of *KIF21A* is very necessary to enhance the credibility of our current study.

## 5 CONCLUSION

In our current research, we demonstrated that *KIF21A* could serve as a potential prognostic biomarker for early‐stage PDAC patients after pancreaticoduodenectomy and patient with a high expression of *KIF21A* would have a poor prognosis. The potential mechanism of *KIF21A* expression level affect patient clinical prognosis outcome might involve in DNA damage and repair, transcription and translation process, post‐translation protein modification, cell cycle, carcinogensis genes and pathways.

## CONFLICT OF INTEREST

There is no conflict of interest disclosed in this study.

## Data Availability

The datasets analyzed during this study are available from the corresponding author on reasonable request.
