# Supplementary figures and images for: Prognostic value of Kinesin‐4 family genes mRNA expression in early‐stage pancreatic ductal adenocarcinoma patients after pancreaticoduodenectomy
Source: Cancer Med. 2019 Sep 6;8(15):6487–502. doi: 10.1002/cam4.2524 (PMC6826000; doi:10.1002/cam4.2524)

**<5.00 E-7**

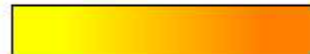

Supplement: Supplementary file 1 [file CAM4-8-6487-s001.pdf]

## Cellular Component

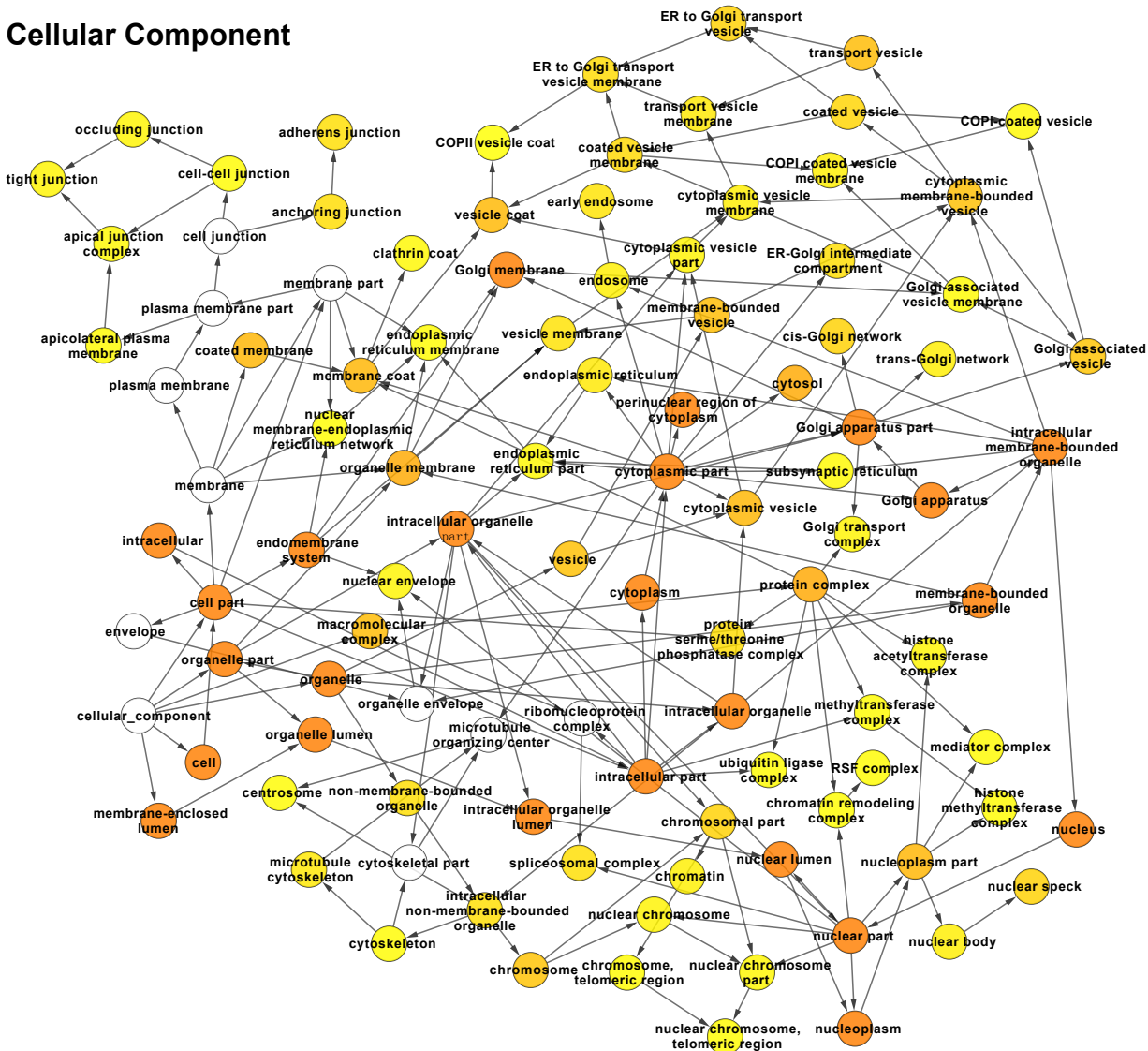

**5.00 E-2**

**<5.00 E-7**

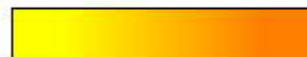

Supplement: Supplementary file 2 [file CAM4-8-6487-s002.pdf]

# Molecular Function

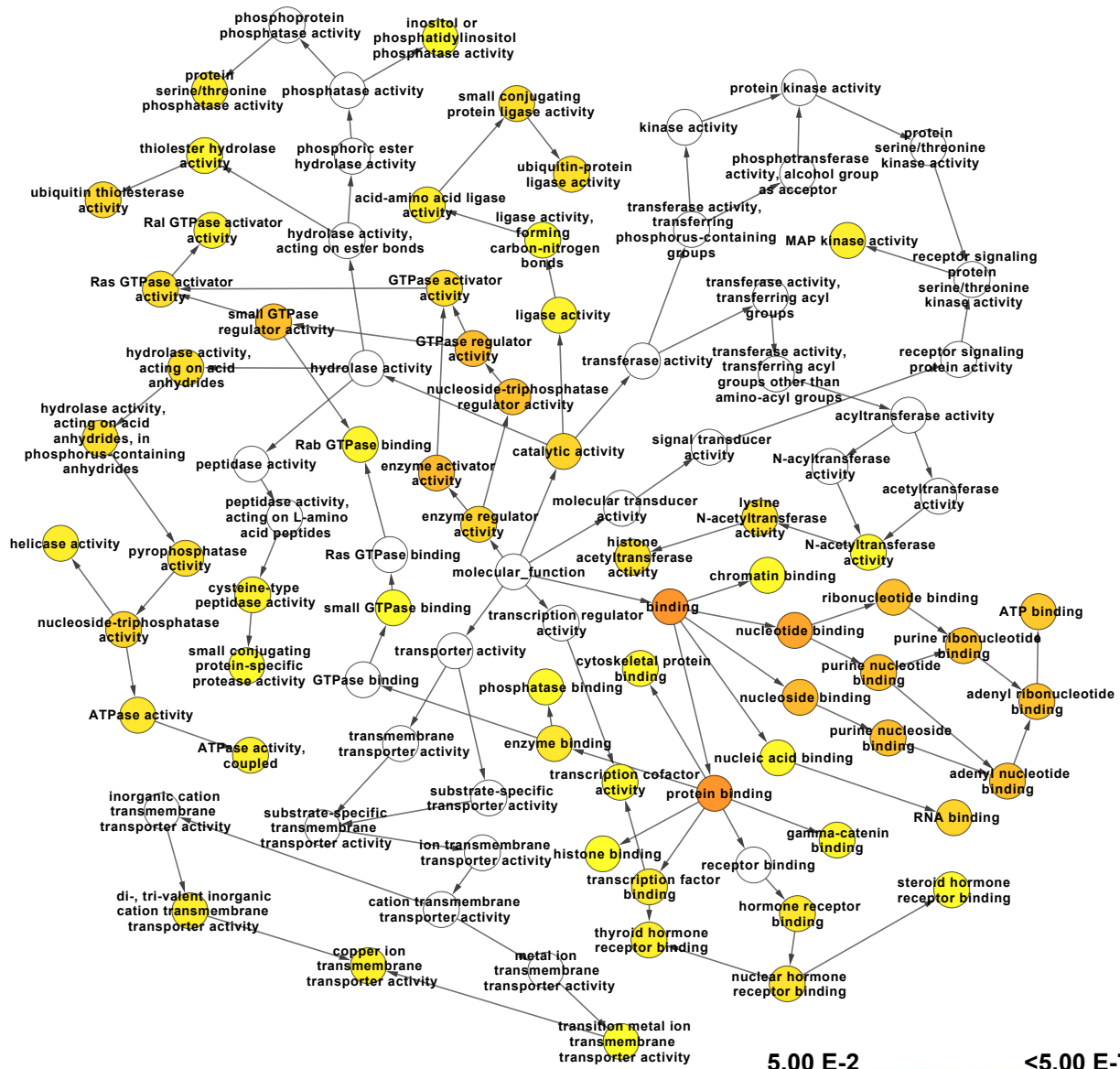

5.00 E-2

<5.00 E-2

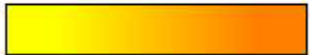

Supplement: Supplementary file 3 [file CAM4-8-6487-s003.pdf]
