# Supplementary material for: Prognostic value of Kinesin‐4 family genes mRNA expression in early‐stage pancreatic ductal adenocarcinoma patients after pancreaticoduodenectomy
Source: Cancer Med. 2019 Sep 6;8(15):6487–502. doi: 10.1002/cam4.2524 (PMC6826000; doi:10.1002/cam4.2524)
Supplement: Supplementary file 4 [file CAM4-8-6487-s004.docx]

**Table S1.** Clinical characteristics of PDAC patients.

| **Variables** | **Patients (n=112)** | **No. of events** | **MST (days)** | **HR (95% CI)** | **Log-rank *P-*value** |
| --- | --- | --- | --- | --- | --- |
| **Age (years)** |  |  |  |  | 0.066 |
| ≤60 | 38 | 20 | 593 | 1 |  |
| ＞60 | 74 | 49 | 485 | 1.636 (0.962-2.780) |  |
| **Gender** |  |  |  |  | 0.523 |
| Female | 53 | 36 | 511 | 1 |  |
| Male | 59 | 33 | 592 | 0.855 (0.529-1.382) |  |
| **Alcohol history﹠** |  |  |  |  | 0.349 |
| No | 43 | 25 | 592 | 1 |  |
| Yes | 61 | 38 | 511 | 1.276 (0.765-2.128) |  |
| **Histologic grade** |  |  |  |  | 0.010 |
| G1+G2 | 80 | 45 | 596 | 1 |  |
| G3+G4 | 32 | 24 | 470 | 1.919 (1.156-3.185) |  |
| **Pathologic stage** |  |  |  |  | 0.943 |
| I | 8 | 4 | 236 | 1 |  |
| II | 104 | 65 | 518 | 1.038 (0.375-2.872) |  |
| **Radical resection＃** |  |  |  |  | 0.009 |
| No | 44 | 29 | 381 | 1 |  |
| Yes | 66 | 39 | 603 | 0.514 (0.310-0.852) |  |
| **Radiation therapy＄** |  |  |  |  | 0.029 |
| No | 70 | 48 | 473 | 1 |  |
| Yes | 30 | 15 | 691 | 0.527 (0.293-0.947) |  |
| **Targeted molecular therapy§** |  |  |  |  | <0.001 |
| No | 29 | 24 | 224 | 1 |  |
| Yes | 73 | 41 | 634 | 0.168 (0.095-0.296) |  |

**Notes**:**﹠**Information of alcohol history was unavailable in 8 patients; **＃**Information of radical resection was unavailable in 2 patients; **＄**Information of radiation therapy was unavailable in 12 patients; **§**Information of targeted molecular therapy was unavailable in 10 patients.

**Abbreviations**: PDAC, pancreatic ductal adenocarcinoma; MST, median survival time; HR, hazard ratio; CI, confidence interval.
